# Risk perceptions and behaviors concerning rural tourism and economic-political drivers of COVID-19 policy in 2020

**DOI:** 10.1371/journal.pone.0299841

**Published:** 2024-04-09

**Authors:** Brandon Lieberthal, Sarah Jackson, Sandra de Urioste-Stone

**Affiliations:** College of Natural Sciences, Forestry, and Agriculture, University of Maine, Orono, ME, United States of America; Fiji National University, FIJI

## Abstract

When COVID-19 was first introduced to the United States, state and local governments enacted a variety of policies intended to mitigate the virulence of the epidemic. At the time, the most effective measures to prevent the spread of COVID-19 included stay-at-home orders, closing of nonessential businesses, and mask mandates. Although it was well known that regions with high population density and cold climates were at the highest risk for disease spread, rural counties that are economically reliant on tourism were incentivized to enact fewer precautions against COVID-19. The uncertainty of the COVID-19 pandemic, the multiple policies to reduce transmission, and the changes in outdoor recreation behavior had a significant impact on rural tourism destinations and management of protected spaces. We utilize fine-scale incidence and demographic data to study the relationship between local economic and political concerns, COVID-19 mitigation measures, and the subsequent severity of outbreaks throughout the continental United States. We also present results from an online survey that measured travel behavior, health risk perceptions, knowledge and experience with COVID-19, and evaluation of destination attributes by 407 out-of-state visitors who traveled to Maine from 2020 to 2021. We synthesize this research to present a narrative on how perceptions of COVID-19 risk and public perceptions of rural tourism put certain communities at greater risk of illness throughout 2020. This research could inform future rural destination management and public health policies to help reduce negative socioeconomic, health and environmental impacts of pandemic-derived changes in travel and outdoor recreation behavior.

## Introduction

In early 2020, when COVID-19 was first introduced to the United States, state and local governments enacted a variety of policies to combat the epidemic [1, 2]. In the absence of vaccines and treatments, the most effective measures to prevent the spread of COVID-19 were non-pharmaceutical solutions including stay-at-home orders, closing of nonessential businesses, social distancing, and the use of masks [3–7]. However, due to a lack of a unified federal response to the epidemic outbreak, the actions taken by governments and individuals to prevent the spread of COVID-19 were highly spatially and temporally variant [8–10]. Although it was well known that regions with high population density, busy airports, and cold climates were at the highest risk for disease spread, we hypothesize that mitigation strategies were instead heavily influenced by other concerns, primarily economic and political [11–14]. For example, rural counties that are economically reliant on tourism and recreation were incentivized to enact fewer precautions against COVID-19 to prevent a reduction in jobs and revenue for the upcoming summer tourism season [15, 16]. In addition, regions with a high electoral margin for President Donald Trump took signals from the federal government that COVID-19 was not a severe crisis and would dissipate on its own [17–21].

In rural tourism destinations, such as those found in Maine, the COVID-19 pandemic presented multifaceted challenges for operations and communities reliant on tourism [22, 23]. The pandemic increased uncertainty to an industry that contributes significantly to national, state, and local economies and livelihoods [24, 25]. From travel restrictions to health and safety protocols, the “new normal” impressed on rural tourism destinations brought forth not only the need to promptly adapt, but further amplified the need to enhance resilience during continued change and vulnerability [26, 27]. COVID-19 impacted short- and long-term planning in rural tourism destinations and continues to influence 1) the development and management of tourism ventures and structures, and 2) the perceptions and behaviors of potential tourists [26–28].

Perceptions and behaviors are integral in the travel decision-making process [29, 30]. The viewpoint a tourist maintains when contemplating a travel-based decision is essential in transferring intangible elements into a tangible tourism experience [22, 29]. During the pandemic, the importance of the socioenvironmental factors influencing travel decisions increased due to the changing nature and timing of planning and actual travel processes [26, 28, 30, 31]. The changing dynamics of when, where, who, and why tourists engaged in travel, along with the fluctuating travel risk perceptions, not only influenced visitation quotas, but also the revenue and vitality of rural tourism destinations [29, 31]. Correspondingly, amid expanding concerns and ambiguity about 1) the probability of traveling, 2) how to complete involved efforts if plausible, and 3) ramifications of such actions (e.g., economic impact, future viability, etc.), understanding the significant, multidimensional role of COVID-19 in the perceptions and behaviors of potential tourists became ever-important, especially for the success of rural tourism destinations [24–26, 31].

Tourist travel decision making, including the assessment of destination attributes, is a multidimensional and complex process that requires further investigation in light of a global health crisis to better understand 1) what factors are important when making travel decisions, 2) how these factors influence travel experiences, and 3) how rural tourism destinations can adapt to better facilitate safe and meaningful tourism experiences [22, 24, 25]. Specifically, prior research has shown that political identity can influence viewpoints and risk perceptions [30–33]. For example, political affiliation has played a role in climate change risk perceptions through influencing people’s beliefs and behaviors [33]. Further, in a health context, political affiliation has impacted the way that individuals receive, synthesize, and interpret messaging surrounding public health and safety protocols [31].

These economic and political hypotheses are commonly held narratives about the trajectory of the COVID-19 outbreak in early 2020 and resulted in mixed effectiveness in controlling the epidemic across the country [34–37]. The most common metric of evaluating the success rates of different states, by the media and the academic community, was to compare their number of infection cases per capita [38–40]. This is a useful metric for determining how severely a population has been affected by the epidemic. However, we posit that during the early epidemic period, the effectiveness of mitigation policy should be measured based on the growth rate of the epidemic, in addition to the raw number of cases [41, 42]. Contrary to popular conception, epidemics in their initial outbreak period do not target only a specific ratio of individuals. Rather, they spread exponentially at a rate dependent on their effective reproduction number, or *R*_*t*_ value [43]. The *R*_*t*_ value is based on the intrinsic reproduction rate of the virus (estimated to be about 2.5 for the original strain) and on local factors such as population density and climate [44–46].

Most importantly, human behavior can increase or decrease *R*_*t*_, and an epidemic can be averted if the *R*_*t*_ value of a community is reduced below one [47–49]. Urban areas of the United States were at much higher risk to suffer more incidence cases per capita than rural areas regardless of their efforts, therefore case number should not be the only method of comparing the relative success of different policies across the country [50, 51]. Incidence rates per capita becomes the best measure of mitigation success when the epidemic has reached its peak and herd immunity suppresses further spread. At this point, the epidemic is expected to infect a ratio of the population equal to $\left(1-\frac{1}{R_{t}}\right)$, providing a direct correlation between the local community’s *R*_*t*_ value and its number of incidence cases per capita [52, 53]. A better method of comparing mitigation success among communities is to compare their evolving *R*_*t*_ values over the course of 2020 [54, 55]. This metric allows us to draw a clear comparison of the relative success rates of different mitigation policies, since a more effective policy would lead to a near-term decrease in *R*_*t*_ and vice versa [56–58]. In this paper we focus specifically on the onset of the pandemic in 2020 so we can analyze the effects of policy and human behavior on *R*_*t*_, without concern for confounding factors such as the arrival of new variants and the distribution of vaccines [59–61].

In this manuscript, we explore the role that political affiliation may have in driving risk perceptions and travel decisions given health and safety concerns during travel (e.g., social distancing, wearing a mask, vaccination rates, etc.). Further, we also aim to detail these roles to support rural tourism destinations through contributing to the enhancement of their overall resiliency. From a statistical perspective, we use publicly available case data to compute the evolving values of *R*_*t*_ for each county in the United States, for each week of 2020 after the COVID-19 virus was introduced. We compare these evolving *R*_*t*_ values to policies enacted by local governments, such as shelter-in-place orders, the closure of nonessential businesses, and the mandatory use of face masks, and we study analytically how economic and political demographics are correlated with *R*_*t*_. From a social science perspective, we conduct a survey of individuals travelling to Maine as a rural tourist destination to study the relationships between political affiliations and risk perceptions of travel behavior during a global pandemic. Through this interdisciplinary approach, we observe and discuss the correlations between the temporally evolving reproduction rate of the COVID-19 epidemic and a county’s rurality, economic reliance on tourism, and support for President Trump, and how these relationships evolve over time.

## Methodology

### Initial state-level analysis

Our first goal was to verify, on a national scale, that there was a statistically significant relationship between state-wide political and economical concerns and their policy response to the COVID-19 pandemic. To that end, we researched the COVID-19 policies of all 50 states and District of Colombia in spring 2020, with a focus on state-wide policies such as shelter-in-place orders and closure of nonessential businesses. We focus our analysis specifically on the year 2020 to eliminate the distribution of vaccines and the arrival of new variants as variables, so that we can precisely study the relationship between human behavior and epidemic severity. We drafted a timeline of each state’s COVID-19 mitigation policies in 2020, and we made note of the duration in days that each state went into lockdown after the virus was introduced to the United States in March. Seven states that had no shelter-in-place or business closure orders were recorded with a lockdown duration of zero. We ran a generalized linear statistical test, using the glm function in R [62], that compared the duration of state lockdowns to factors that are correlated with COVID-19 risk of infectiousness or fatality (population density, mean spring temperature, and mean age), along with two predictors that indicate potential economic and political influences [14, 63, 64]. Specifically, we compiled each state’s percent GDP dependence on the tourism industry in 2019 from their Department of Tourism or equivalent department reports, and we recorded their net support for President Trump based on his margin of victory in the 2020 presidential election.

### National incidence curve analysis

**Fig 1:**
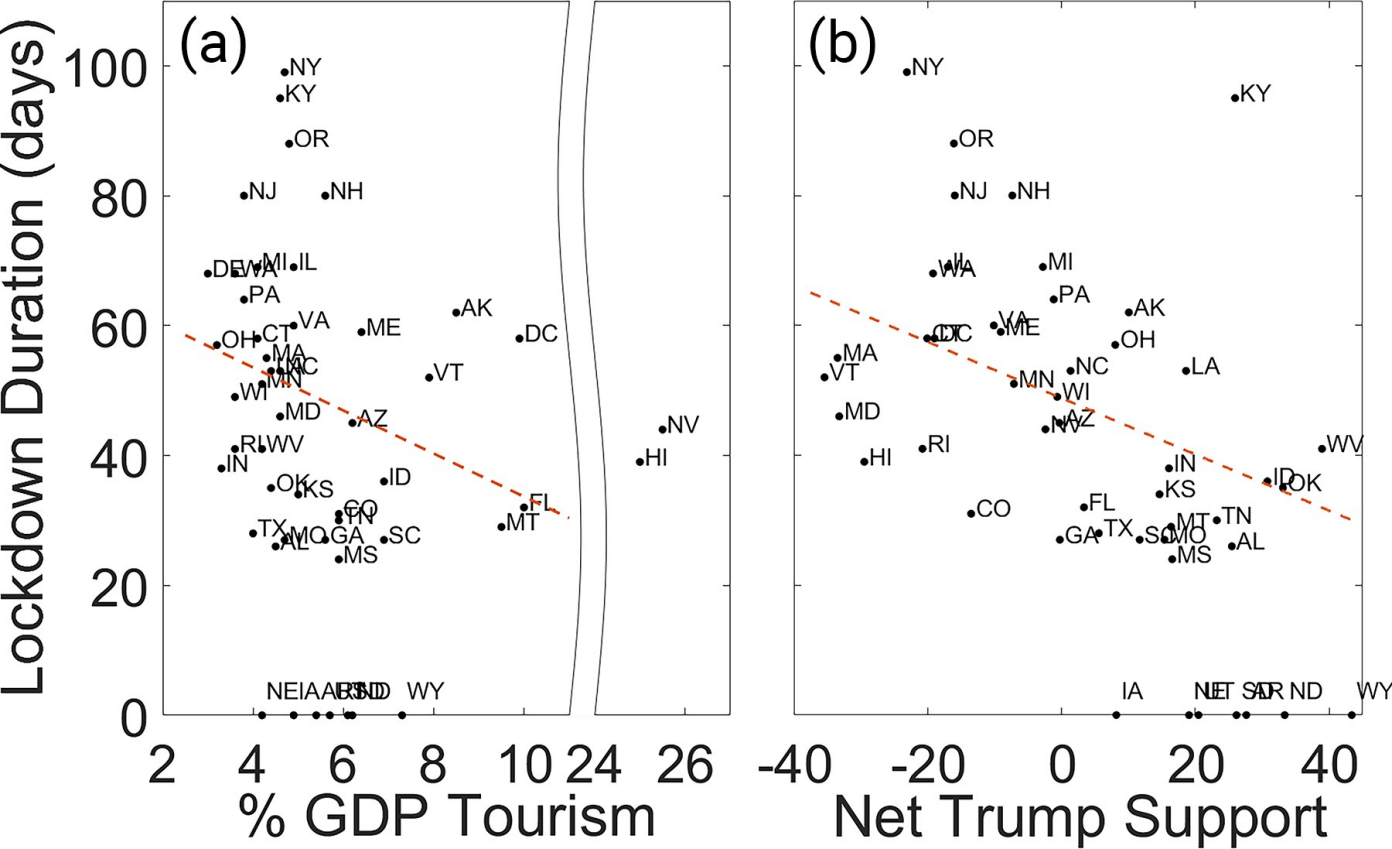
(a) A bar graph of the number of reported cases of COVID-19 in Kennebec County, Maine, throughout 2020. The raw case data was compiled from a variety of sources by the New York Times. The data is cleaned, first by averaging the data over a seven-day period (dotted line), then by assuming an 8-day lag between the onset of infection and the appearance of symptoms (solid line). (b) The effective reproduction number (*R*_*t*_) in Kennebec county, computed from the cleaned case data using the Bettencourt and Ribeiro method. The shaded region represents a 90% confidence interval.

We collected COVID-19 incidence case data for each of the 3143 counties in the United States from the New York Times database [65]. For each county, we computed a time series of the number of new reported incidence cases daily in the year 2020 (Fig 1A). This case data is subject to several reporting biases, including the tendency of hospitals to report cases to local authorities on weekends, the time duration between the initial infection and the onset of symptoms, and unreported asymptomatic cases. Therefore, the data require considerable cleaning before they can be used to compute *R*_*t*_.

Our data cleaning process was as follows. First, we smoothed the incidence case time series for each county over a 7-day moving average to eliminate the bias towards weekend reporting. Next, we assumed that there is on average an eight-day duration between the time an individual is first infected with the COVID-19 virus and when their symptoms are reported to local authorities and adjusted the time series accordingly [66]. Because we use Maine as a case study in this paper, an example of this process for Kennebec County, Maine, is shown in Fig 1A. We calculated the *R*_*t*_ value for each week in each county using a modified Bettencourt and Ribeiro method [67]. For this method, we assumed that the number of new cases each day within a county follows a Poisson distribution

$$L(\lambda |k)=\frac{\lambda^{k}e^{-\lambda }}{k!}$$

where *λ* is the arrival rate of new infection cases. We used the previous two weeks of incidence data to estimate the most likely value of *λ*. With an estimate for *λ*, we could calculate the *R*_*t*_ value using the equation

$$\lambda =k_{t-1}e^{\gamma (R_{t}-1)}$$

where *k*_*t*−1_ is the number of infection cases at the previous time interval, and *γ* is the serial interval (the time duration from illness onset in the primary case to illness onset in the secondary case) of COVID-19, estimated to be about four days [68].

This analysis results in a time series of the evolving *R*_*t*_ value for each county in 2020. To mitigate noisy data, we excluded counties with less than 100 total cases, and we smoothed the time series using spline interpolation. An example of an *R*_*t*_ time series for Kennebec County is given in Fig 1B. An *R*_*t*_ value above 1 indicates that the number of incidence cases were increasing, and an *R*_*t*_ value below 1 indicates that cases were declining. This example also highlights the two types of outbreaks that may occur. In April, the *R*_*t*_ value increased to 3 for a short period of time, resulting in a relatively small wave of cases. In October, the *R*_*t*_ value ranged from 1–2 and was sustained for several months, resulting in a much longer and more severe wave. In general, the total number of cases throughout the course of a wave is dependent not just on the value of *R*_*t*_ but also the length of time it is elevated above 1.

At each week of 2020, we ran a statistical test on the *R*_*t*_ value of each county with at least 100 incidence cases, using a variety of predictor variables that include climate, demographics, and population density, and political and economic indicators, using the glm function in R on the assumption that *R*_*t*_ follows a Poisson distribution (Table 1) [69]. We ran a Pearson correlation analysis to ensure that none of the predictor variables were cross-correlated with a correlation coefficient greater than 0.6. We also ensured that at each week of the experiment, there was no detected variable endogeneity with a Wu-Hausman p-value of at least 0.9. For each predictor variable, we divided the estimated value coefficient by its standard error to obtain a dimensionless t-test score of correlation. According to the cumulative density function of the t-distribution, a t-test score of about 1.5 or higher indicates a strong positive correlation between the given predictor variable and the *R*_*t*_ value, and a score of -1.5 or lower indicates a strong negative correlation, with a p-value of 0.1. A t-test score close to zero indicates no strong positive or negative correlation. By plotting the t-test score of a predictor variable as it evolves over time, we could measure quantitively the moments in time that these variables were strong predictors of a COVID-19 epidemic. We focused on three variables in particular that were relevant to our hypotheses: the rurality of the county, the state’s economic reliance on tourism, and the county’s support for President Trump. It is important to note that this is not a time series analysis, as we tested each week of the experiment as an independent cross-section in time. Therefore, this research does not make any conclusions on causality between the predictor variables and *R*_*t*_ value, just whether there exists a correlation between them.

**Table 1:** Statistical summary of a linear model comparing the duration of lockdown orders of all 50 states and District of Colombia in early 2020 compared to their economic reliance on tourism, net support for President Trump, population density, mean temperature in spring 2020, and mean age of the population.

	Estimate	Std. Error	z value	p value
(Intercept)	3.88E+00	4.62E-01	8.39	<2E-16
% GDP Tourism	-3.98E-02	1.27E-02	-3.12	0.00179
Net Trump Support	-8.06E-03	9.73E-04	-8.29	<2E-16
Population Density	-8.17E-05	3.78E-05	-2.16	0.0309
Mean Temperature	5.44E-03	3.64E-03	1.49	0.135
Mean Age	-7.62E-03	1.10E-02	-0.70	0.487

We also sought to test whether there was a statistically significant geographic distribution in *R*_*t*_ values. In addition to the t-test analysis, we also tested for spatial autocorrelation through Geographically Weighted Regression and by measuring Moran’s I at each week of 2020. By both metrics, no statistically significant spatial autocorrelation was detected. In other words, when controlling for demographics, *R*_*t*_ values were not significantly correlated among counties geographically adjacent to each other.

### Maine incidence curve analysis

We ran a similar analysis focusing specifically on the state of Maine. We chose Maine as a case study because despite Maine’s racial homogeneity and high average age, it is unusually diverse in terms of rurality, tourism, and political leanings. Maine is divided into sixteen counties by the state legislature and eight tourism zones by the Maine Department of Economic and Community Development, which overlap with county borders [70]. The state produces annual economic reports for each tourism zone, which we interpolated over the counties with a weighted population algorithm in ArcGIS Pro 2.8.1. The tourism industry comprised 17% of Maine’s jobs in 2019, although among individual counties this ranged from 3% in rural Somerset County to 22% in Hancock County, the site of Bar Harbor and Acadia National Park [23].

The advantage of a state-level analysis, as opposed to the federal-level, is that we can directly study the correlation between the governor’s policy decisions and the variance in *R*_*t*_ values across the counties. Maine Governor Janet Mills declared a state of emergency on April 2, 2020 and closed all nonessential businesses [71]. Cruise ship ports were closed one week later, on April 8. Businesses were reopened on May 31, and the ports were reopened on July 1, although cruise ship traffic did not resume until 2021. Throughout the tourism season, compared to 2019 the total number of visitors was reduced from 16 million to 12 million individuals, and revenue was reduced from $6.5 billion to $4.8 billion [23].

### Survey methodology

A Qualtrics online survey of Maine out-of-state visitors was conducted in 2021 to understand travel decisions and behaviors while traveling to Maine during the pandemic; a total of 407 quality responses were acquired through a panel managed by Qualtrics. This panel included the voluntary participation of individuals who were at least 18 years of age and identified as out-of-state visitors. Specifically, participants from Massachusetts, New Hampshire, New York, Connecticut, Vermont, Rhode Island, Pennsylvania, North Carolina, and Florida were involved within the panel survey process. The online survey inquired about travel behavior and motivations, perceptions and experience regarding COVID-19, and sociodemographics. For analysis procedures, participants were grouped based on their identified political affiliation: conservative (N = 93), independent (N = 117), or liberal (N = 113; Table 2). To evaluate associations concerning political affiliation, chi-square analyses were completed using SPSS (Version 28). Further, one-way ANOVA analyses were also implemented to determine statistically significant differences amongst groups. A Games-Howell post hoc test was used to further compare across political affiliation categories.

**Table 2:** Reported demographic percentages for all respondents and for those who identified with a specific political affiliation (i.e., conservative, independent, and liberal).

		All Respondents	Conservative	Independent	Liberal
		(N = 407)	(N = 93)	(N = 117)	(N = 113)
*Age (Years)*	18–24	9.2%	4.4%	10.6%	11.8%
	25–34	33.0%	33.0%	31.0%	34.5%
	35–44	27.9%	28.6%	26.5%	29.1%
	45–54	15.9%	14.3%	19.5%	13.6%
	55–64	7.3%	8.8%	8.0%	5.5%
	Above 64	6.7%	11.0%	4.4%	5.5%
*Race and Ethnicity* *	American Indian or Alaska Native	2.3%	2.9%	1.7%	2.4%
	Asian	4.0%	2.0%	5.0%	4.8%
	Black or African American	11.8%	9.8%	11.6%	13.7%
	Hispanic or Latin American	7.8%	6.9%	6.6%	9.7%
	Native Hawaiian or Pacific Islander	0.6%	1.0%	0.0%	0.8%
	White	73.5%	77.5%	75.2%	68.5%
*Highest level of education*	Some high school	3.7%	2.2%	5.1%	3.5%
	High school diploma or equivalent	28.8%	36.6%	32.5%	18.6%
	Some college, no degree	22.3%	22.6%	20.5%	23.9%
	Associate’s degree	15.8%	12.9%	19.7%	14.2%
	Bachelor’s degree	21.7%	15.1%	17.9%	31.0%
	Master’s degree	6.2%	6.5%	4.3%	8.0%
	Doctorate degree	1.5%	4.3%	0.0%	0.9%
*Sex assigned at birth*	Male	38.7%	48.4%	38.5%	31.0%
	Female	61.0%	50.5%	61.5%	69.0%
	Prefer not to reply	0.3%	1.1%	0.0%	0.0%
*Annual household income*	Less than $24,999	16.4%	18.3%	15.4%	15.9%
	$25,000 to $34,999	14.9%	9.7%	17.9%	15.9%
	$35,000 to $49,999	19.8%	22.6%	22.2%	15.0%
	$50,000 to $74,999	23.5%	24.7%	21.4%	24.8%
	$75,000 to $99,999	9.9%	10.8%	7.7%	11.5%
	$100,000 to $149,999	10.8%	8.6%	11.1%	12.4%
	$150,000 to $200,000	3.4%	3.2%	4.3%	2.7%
	Greater than $200,000	1.2%	2.2%	0.0%	1.8%

*Percentages based on option to “select all that apply” for specified question.

The study obtained ethical approval from the University of Maine (# 2021_09_07) Internal Review Board. Survey participants were adults 18 years and over, who received a written consent prior to agreeing to complete the online survey.

## Results

### Initial state-level analysis

**Fig 2:**
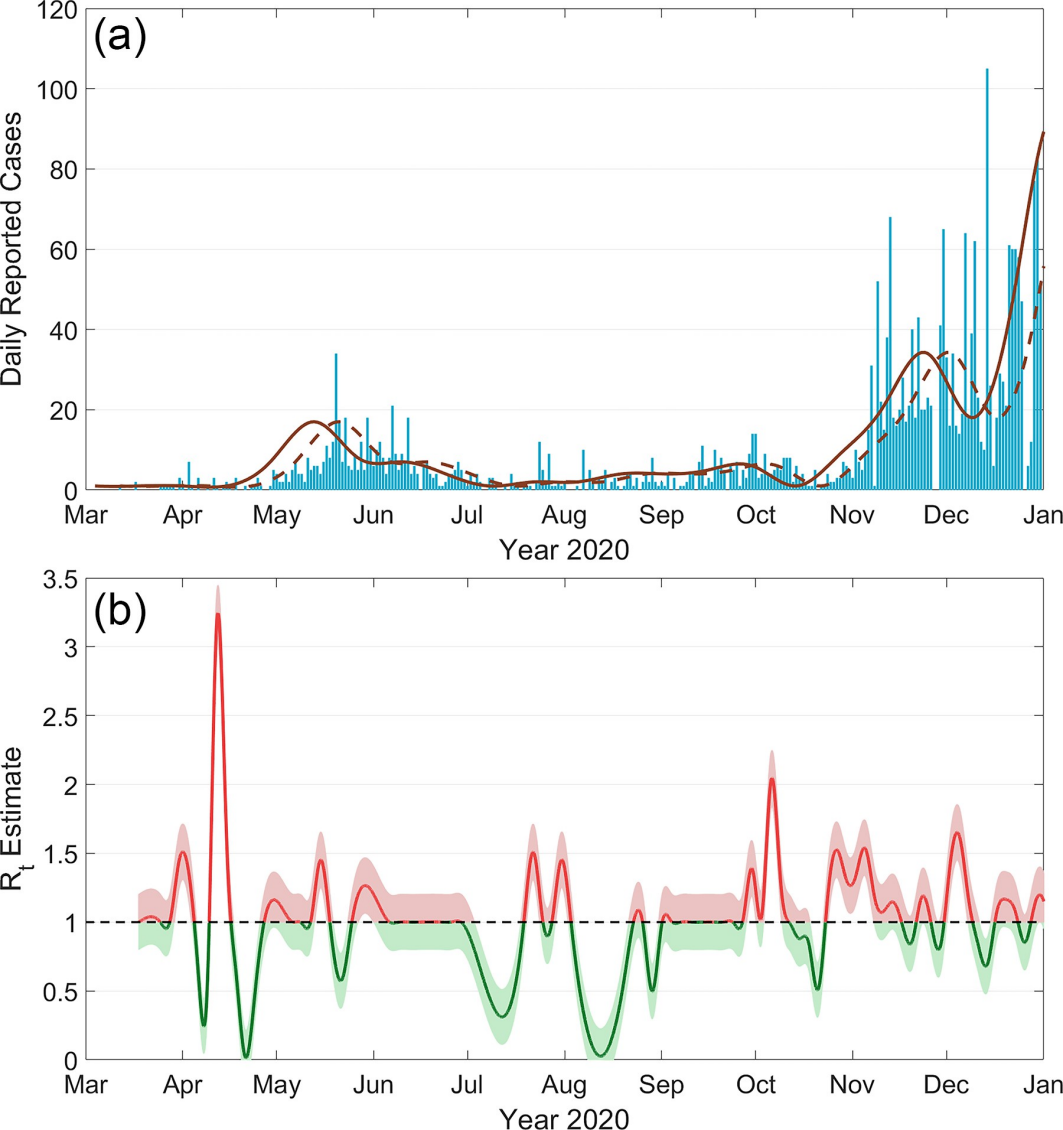
Scatter plots showing the duration of lockdown orders of all 50 states and District of Colombia, compared to their % GDP tourism in 2019 and margin of incumbent President Trump’s election victory in November 2020. Two states (Hawaii and Nevada) are considered outliers and are not included in the linear model fit, and two states (California and New Mexico) had lockdown durations that lie far above the upper boundary of the plots. Seven states which did not enact lockdown orders (Arkansas, Iowa, Nebraska, North Dakota, South Dakota, Utah, and Wyoming) are recorded with a duration of 0 days.

The results of our analysis, which statistically compare the duration of state lockdowns during the initial COVID-19 outbreak, validate our hypotheses that policy was heavily influenced by economic and political factors (Fig 2). The two predictor variables most strongly correlated with the duration of state lockdowns were the state’s GDP dependence on tourism (z = -3.1, p < 0.01) and net support for President Trump (z = 8.3, p < 0.01). Other predictors such as population density, mean age, and mean temperature had much weaker correlations with lockdown duration (Table 1). This indicates that economic and political motivations were more strongly correlated with COVID-19 mitigation policy than actual risk factors of infectiousness or fatality, and this result was a key motivator for the rest of the research conducted in this paper. We note that this model is not intended to be a complete analysis and is only intended to provide motivation for the research performed in this manuscript. Although there may be other variables influencing lockdown policy that we did not consider, a Wu-Hausman test indicated no concerns with predictor endogeneity (H = 0.048, df = 2,37, p = 0.95).

### COVID-19 incidence curve analysis

**Fig 3:**
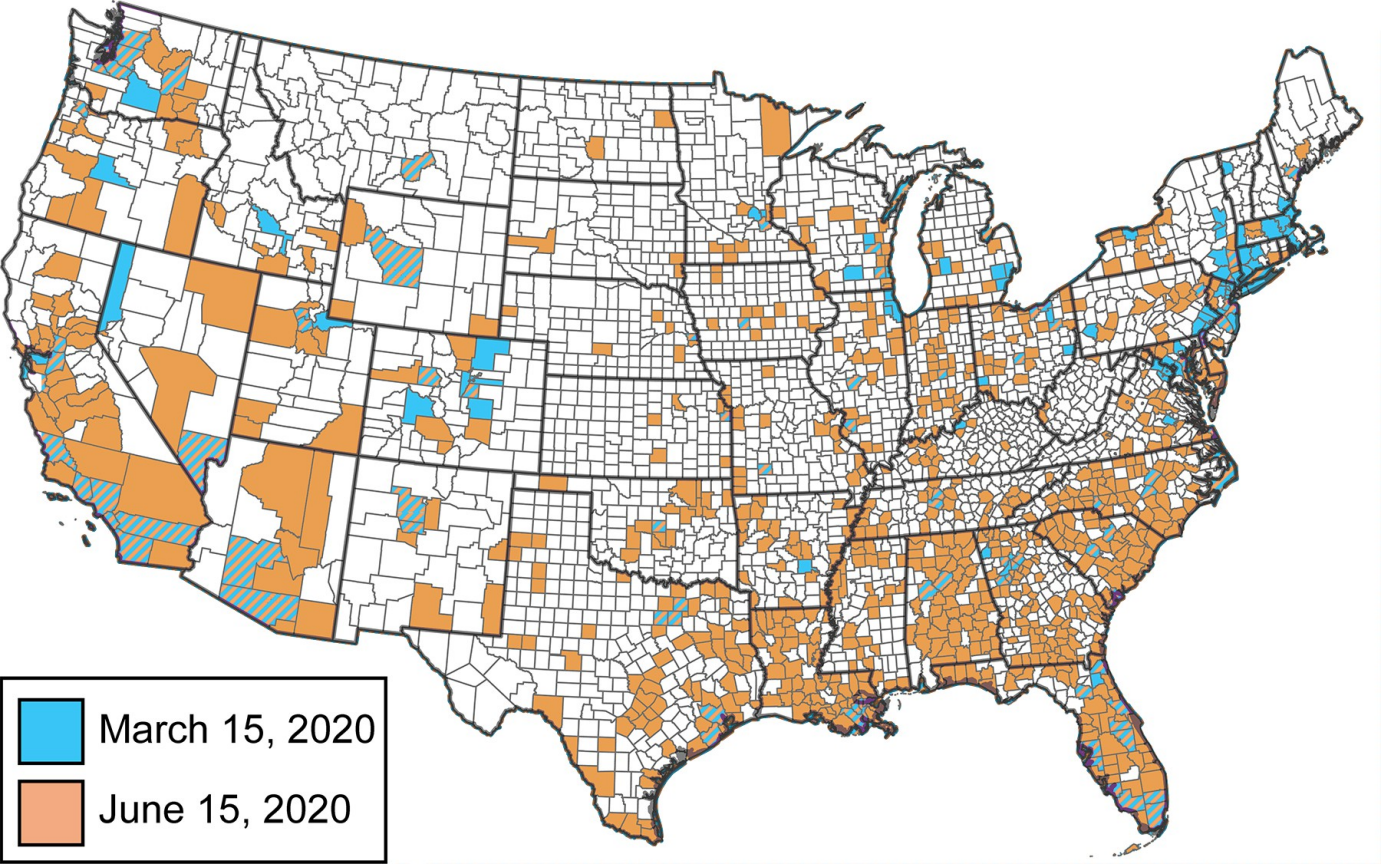
The counties in the continental United States experiencing an epidemic (*R*_*t*_>1) in March 15 (blue) and June 15 (orange), 2020. The counties that are striped blue and orange were experiencing an epidemic on both dates. This map is adapted from the US County Map provided by the U.S. Geological Survey and is available in the public domain [102].

**Fig 4:**
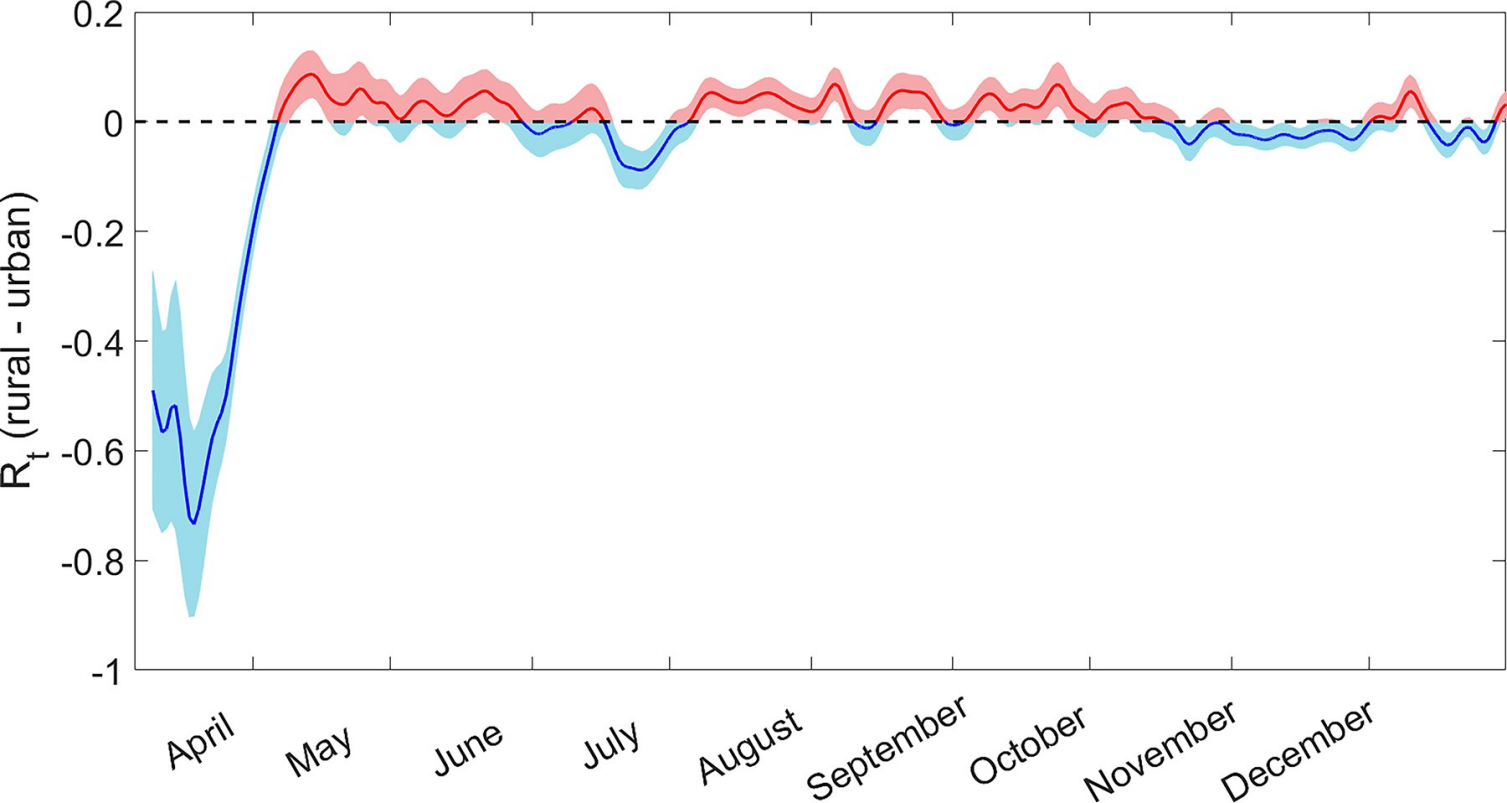
The difference in effective reproduction number *R*_*t*_ between all rural counties and all urban counties in the United States, as defined by the American Community Survey. A positive value indicates that COVID-19 was more infectious in rural than urban counties, and vice versa. The shaded region represents a 90% confidence interval.

The following results are based on the time evolving and spatially varying value of *R*_*t*_, computed on the county-level, as defined in the Methods section. During the initial outbreak in March 2020, epidemic hotspots with *R*_*t*_ values greater than one were primarily located in urban centers along the coasts and in the Midwest (Fig 3). In general, the *R*_*t*_ value among all urban counties was about 0.75 higher than the *R*_*t*_ value among all rural counties (Fig 4). This resulted in a short but significant wave of cases in urban areas while rural areas were less affected. In the summer of 2020, although urban counties in California, Washington, and Florida were still experiencing outbreaks, most other urban areas had *R*_*t*_ values less than 1. Instead, COVID-19 hot-spots were distributed geographically, primarily in the plains west and deep south. For most of the period between May and October, *R*_*t*_ values were less than 0.1 higher in rural than urban counties. Although the differences in *R*_*t*_ were small, they were sustained over several months, and by September the number of incidence cases per capita in rural America surpassed those in urban America, despite urban America’s much higher population density.

**Fig 5:**
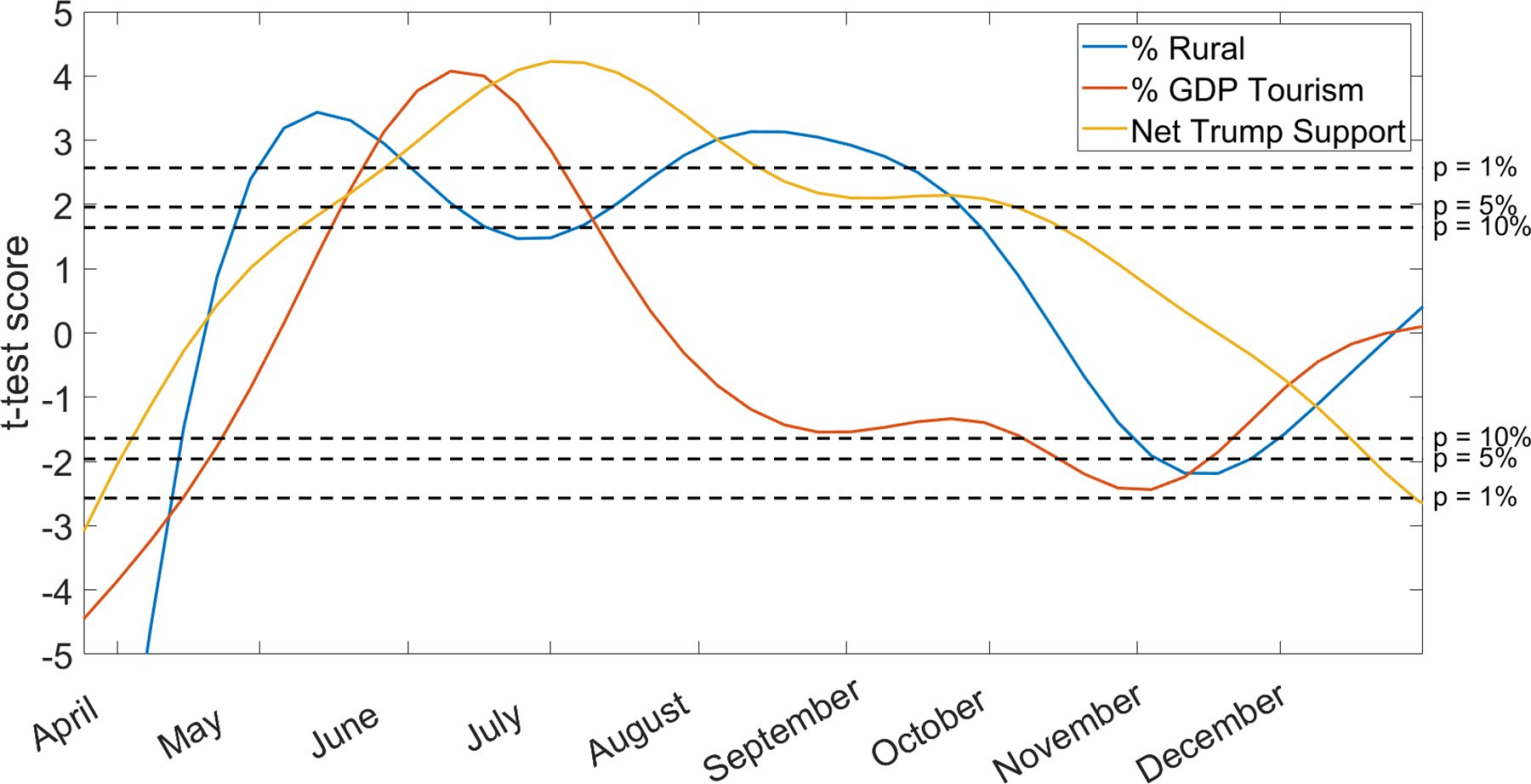
The results of a linear model for each week of 2020, comparing the *R*_*t*_ value in all 3143 counties in the United States to the following predictor variables: % Population that lives in a rural area, % GDP reliance on tourism in 2019, net support for President Trump, and the other predictor variables listed in Table 1. A high positive t-test score (coefficient estimate / standard error) indicates a strong positive correlation between the given predictor variable and the *R*_*t*_ value, and a low negative t-test score indicates a strong negative correlation. A t-test score close to zero indicates no strong positive or negative correlation. The thresholds for a 10%, 5%, and 1% p-value are marked on the figure.

Throughout the first half of 2020, a strong positive correlation emerged between county rurality, economic reliance on tourism, and support for President Trump with the value of *R*_*t*_ (Table 3, Fig 5). In March and early April, *R*_*t*_ values were significantly higher in counties with low rurality and low tourism (p < 0.01), and correlation between *R*_*t*_ and Trump support was weak. This relationship began to flip in late May, and throughout the summer *R*_*t*_ becomes statistically higher in rural counties with large tourism industries and high Trump support (p < 0.01). The strong statistical relationship between tourism and *R*_*t*_ ends in the fall, but rurality and Trump support remain strong predictors until the winter. By the end of 2020, the t-test scores for all three predictors are at values close to zero.

**Table 3:** A list of predictor variables used in the statistical test of *R*_*t*_ value by county, along with their sources.

Predictor	Unit	Source
Rural	% population living in a designated rural zone	Census
Tourism	% GDP reliance on tourism in 2019	State Departments of Tourism
Trump	Net popular support for President Trump	2020 Presidential Election Results
Population density	Mean population density	Census
Mean income	Mean annual income of adult population	American Community Survey
Temperature	Mean monthly temperature	CHELSA
Precipitation	Total monthly precipitation	CHELSA
Religion	% population that identify as religious	American Community Survey
Health Insurance	% population with health insurance	American Community Survey
Education	% population with a 4-year college degree	American Community Survey
Lockdown	= 1 if state was in lockdown that week, = 0 otherwise	New York Times
Latitude	Deg. Latitude of centroid	Census
Longitude	Deg. Longitude of centroid	Census

**Fig 6:**
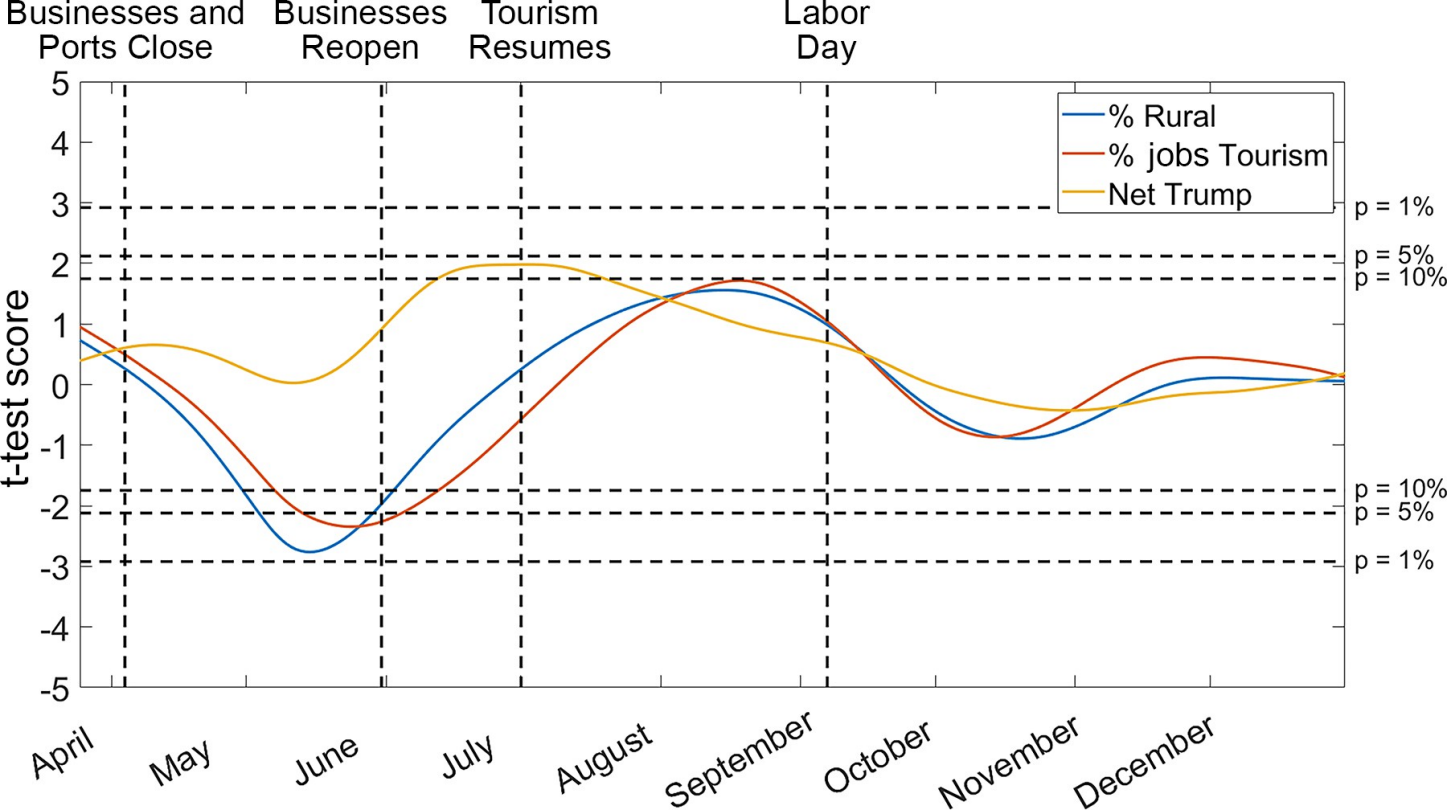
This figure is analogous to Fig 5, focused on the 16 counties of Maine. Note that economic reliance on tourism is measured in terms of the percent of jobs, rather than percent of GDP, due to the availability of fine scale data. Also note that because of the reduced degrees of freedom, the threshold for a 1% p-value is slightly higher than in Fig 5. Key dates in Maine’s COVID-19 response and summer tourism season are marked on the figure.

A similar dynamic took place in the analysis focusing on Maine (Fig 6). Since there are now only 16 data points instead of over 3000, the t-test scores are less extreme and we only observe p-values as low as 0.1. However, the same pattern emerges as on the federal level, but delayed in time. As opposed to many regions of the county whose tourism seasons began in May, tourism in Maine was shut down until July. For the rest of the summer, *R*_*t*_ values were highest in counties with rural counties with large tourism industries and high Trump support (p = 0.1).

### Visitor profile and group characteristics

Respondents primarily self-identified as 25 to 34 years of age (33.0%), female (61.0%), White (73.5%), acquired a high school diploma or equivalent (28.8%), and reported an annual household income of $50,000 to $74,999 (23.5%; Table 2). Group differences were found concerning highest level of education and annual household income.

Conservatives were predominantly white (77.5%), obtained a high school diploma or equivalent (36.6%), and reported an annual household income of $50,000 to $74,999 (24.7%). For those who identified as independent, most were white (75.2%), with a high school diploma or equivalent (32.5%) and reported receiving an annual household income equivalent to $35,000 to $49,999 (22.2%). In contrast, those who identified as liberal were predominantly white (68.5%), expressed obtaining a bachelor’s degree (31.0%), and reported an annual household income within the range of $50,000 to $74,999 (24.8%).

### The interaction of political affiliation and COVID-19 perceptions and behaviors

Perceptions on the COVID-19 pandemic varied across all groups (Table 4). There were statistically significant differences between conservatives and independents and liberals when assessing the severity (*F* = 9.8, p = 0.005) and potential health implications (*F* = 9.9, p < 0.001) of COVID-19. Associations were also found between groups and opinions regarding scientists’ understanding of COVID-19 (χ^2^ = 36.0, p < 0.001) and governmental strategies to address the pandemic (χ^2^ = 28.0, p < 0.001). Specifically, conservatives and liberals showed statistically significant differences in terms of their viewpoint(s) of scientists with liberals having greater trust in scientists’ level of understanding about the pandemic (*F* = 18.3, p < 0.001) and their own understanding about the government’s pandemic strategy (*F* = 9.9, p < 0.001).

**Table 4:** Percent agreement concerning COVID-19 based on participants identified political affiliation (i.e., conservative (N = 93), independent (N = 117), and liberal (N = 113)).

	Political affiliation	Strongly disagree	Disagree	Neither disagree nor agree	Agree	Strongly agree	Chi square (χ^2^)	*df*	Sig	ANOVA *F*
*Getting sick with COVID-19 can be serious*	Conservative^ab^	0.9%	2.8%	5.9%	10.8%	8.4%	27.3	8	<0.001	9.8
	Independent^*a*^	0.9%	0.9%	3.4%	10.5%	20.4%				
	Liberal^b^	0.9%	1.9%	2.2%	9.6%	20.4%				
*I am personally worried about COVID-19*	Conservative^b^	5.0%	3.4%	7.1%	8.4%	4.7%	22.0	8	0.005	9.9
	Independent	4.3%	4.0%	6.2%	14.9%	6.8%				
	Liberal^b^	0.9%	2.5%	6.8%	14.3%	10.6%				
*I feel that I understand the government’s strategy to deal with the COVID-19 pandemic*	Conservative^b^	5.3%	4.6%	9.3%	6.5%	3.1%	28.0	8	<0.001	9.9
	Independent	3.7%	3.4%	11.5%	10.2%	7.4%				
	Liberal^b^	2.8%	3.4%	5.3%	14.9%	8.7%				
*I think that scientists have a good understanding of COVID-19*	Conservative^b^	6.2%	5.6%	7.1%	6.8%	3.1%	36.0	8	<0.001	18.3
	Independent	3.7%	5.0%	9.6%	10.2%	7.7%				
	Liberal^b^	1.9%	3.1%	5.6%	11.5%	13.0%				
*I think it is important to do something (like wear a mask) for the benefit of others even if it may come at a cost to me personally*	Conservative^b^	3.4%	3.7%	5.3%	10.2%	6.2%	43.2	8	<0.001	23.0
	Independent^c^	1.9%	2.5%	6.5%	12.4%	13.0%				
	Liberal^bc^	0.3%	0.3%	2.5%	13.0%	18.9%				
*I think it is important to do something for the benefit of others even if it may not be the popular choice*	Conservative^ab^	3.4%	2.5%	8.4%	9.9%	4.6%	43.5	8	<0.001	21.4
	Independent^a^	1.5%	0.3%	6.8%	14.2%	13.3%				
	Liberal^b^	0.6%	0.9%	5.0%	10.5%	18.0%				

^abc^Statistical difference at a p < 0.001 based on post hoc analyses.

Further, differences in behavioral intent were detected in terms of actions that benefit others, such as wearing a mask even if it poses a form of personal cost (χ^2^ = 43.2, p < 0.001) or is divergent to the popular choice (χ^2^ = 43.5, p < 0.001). Statistically significant differences were found across groups, with conservatives having higher levels of disagreement than liberals, and liberals having higher levels of agreement than independents (*F* = 23.0, p < 0.001) about conducting behaviors that benefit others despite personal costs. Contrastingly, conservatives were less likely than independents or liberals to engage in COVID-19 protective behaviors if they were not the popular choice (*F* = 21.4, p < 0.001).

### Experience and trust

Concerning personal experience with COVID-19, associations were found across political affiliation and traveler’s experiences with the pandemic (Table 5). Associations were found between political affiliation and COVID-19 vaccination (χ^2^ = 16.1, p = 0.003); those who identified as liberal reported a higher likelihood to receive or plan to receive the COVID-19 vaccine, with independents displaying the second highest, and conservatives conveying the lowest.

**Table 5:** Opinions concerning experiences with COVID-19 based on participants identified political affiliation (i.e., conservative (N = 93), independent (N = 117), and liberal (N = 113)).

	Political affiliation	No	Yes	Chi square (χ^2^)	*df*	Sig	ANOVA *F*
*I have had*, *or think I may have had COVID-19*	Conservative	18.9%	8.0%	1.1	4	0.895	0.2
	Independent	24.1%	9.6%				
	Liberal	22.6%	10.8%				
*I have received the COVID-19 vaccine or plan to receive the COVID-19 vaccine*	Conservative	12.7%	15.5%	16.1	4	0.003	1.6
	Independent	11.8%	23.2%				
	Liberal	6.5%	27.6%				
*One or more of my friends has had*, *or thinks they have had*, *COVID-19*	Conservative	10.5%	17.3%	2.6	4	0.624	0.7
	Independent	12.4%	21.4%				
	Liberal	10.2%	22.9%				
*Someone in my immediate family (e*.*g*., *parents*, *siblings) has had*, *or thinks they have had*, *COVID-19*	Conservative	12.7%	15.2%	3.1	4	0.534	0.9
	Independent	20.1%	15.5%				
	Liberal	16.7%	17.3%				
*Someone in my extended family (e*.*g*., *cousins*, *uncles/aunts*, *etc*.*) has had*, *or thinks they have had*, *COVID-19*	Conservative	11.8%	15.2%	2.7	4	0.607	0.2
	Independent	16.1%	18.3%				
	Liberal	12.1%	21.1%				
*I personally know someone who has died of COVID-19*	Conservative	15.2%	12.4%	7.8	4	0.100	0.1
	Independent	22.3%	11.8%				
	Liberal	15.8%	17.6%				

Associations were also reflected regarding the level of trust held in information sources by political affiliation group (Table 6) in consideration of the accuracy of COVID-19 information provided by scientists (χ^2^ = 43.6, p < 0.001), the government (χ^2^ = 35.7, p < 0.001), and news media (χ^2^ = 26.1, p = 0.001). Conservatives were less likely than liberals, and independents less inclined than liberals to trust scientists to convey accurate information about the pandemic (*F* = 22.0, p < 0.001). Concerning the government, statistically significant differences were found between conservatives and liberals, with conservatives having less trust in the government to provide accurate information on COVID-19 (*F* = 16.7, p < 0.001). Further, when considering news media, there were significant differences across groups, with conservatives being less inclined than liberals to trust this source when it came to information about the pandemic (*F* = 8.5, p = 0.001). No significant differences were found across groups in terms of their trust towards social media platforms, with low levels of trust conveyed across all political affiliation groups.

**Table 6:** Percent agreement regarding trust in information sources based on participants identified political affiliation (i.e., conservative (N = 93), independent (N = 117), and liberal (N = 113)).

	Political affiliation	Strongly disagree	Disagree	Neither Disagree nor agree	Agree	Strongly agree	Chi square (χ^2^)	*df*	Sig	ANOVA *F*
*I trust scientists to provide accurate information on COVID-19*	Conservative^a^	5.0%	6.5%	7.4%	6.8%	3.1%	43.6	8	<0.001	22.0
	Independent^b^	4.0%	4.6%	10.5%	11.8%	5.3%				
	Liberal^ab^	1.2%	2.2%	6.2%	13.3%	12.1%				
*I trust the government to share accurate information on COVID-19*	Conservative^a^	10.2%	4.0%	6.5%	5.6%	2.5%	35.7	8	<0.001	16.7
	Independent	6.5%	6.5%	9.0%	10.2%	4.0%				
	Liberal^a^	3.4%	2.5%	8.4%	13.3%	7.4%				
*I trust family and friends to provide accurate information on COVID-19*	Conservative	1.2%	2.5%	11.8%	9.6%	3.7%	8.1	8	0.419	0.6
	Independent	0.9%	3.1%	12.7%	14.2%	5.3%				
	Liberal	1.5%	4.3%	8.4%	14.9%	5.9%				
*I trust news media to provide accurate information on COVID-19*	Conservative^a^	9.9%	5.3%	5.9%	6.2%	1.5%	26.1	8	0.001	8.5
	Independent	5.9%	7.1%	9.0%	9.6%	4.6%				
	Liberal^a^	3.7%	4.6%	12.1%	10.8%	3.7%				
*I trust social media to provide accurate information on COVID-19*	Conservative	9.3%	5.3%	6.2%	6.5%	1.5%	9.7	8	0.287	0.6
	Independent	8.4%	8.7%	10.8%	6.5%	1.9%				
	Liberal	5.9%	9.0%	11.1%	6.5%	2.5%				

^ab^Statistical difference at a p < 0.001 based on post hoc analyses.

### Travel to destinations

Concerning traveling and COVID-19, associations were found amongst those who identified as conservative, independent, or liberal and their travel behavior (Table 7). Primarily, associations were reported regarding the level of concern maintained when planning or implementing travel procedures (χ^2^ = 15.6, p = 0.049). All political affiliation groups were likely to experience concern when making travel-based decisions, but conservatives were less likely to express worry overall. Both independents and liberals were more likely to reflect concern when making their decision to engage in travel behaviors.

**Table 7:** Percent agreement regarding COVID-19 and traveling based on participants identified political affiliation (i.e., conservative (N = 93), independent (N = 117), and liberal (N = 113)).

	Political affiliation	Strongly disagree	Disagree	Neither disagree nor agree	Agree	Strongly agree	Chi square (χ^2^)	*df*	*Sig*	ANOVA F
*I am not concerned with safety when choosing to travel to destinations*	Conservative	5.3%	6.5%	6.5%	5.6%	5.0%	15.6	8	0.049	3.7
	Independent	8.7%	9.0%	9.3%	7.1%	2.2%				
	Liberal	9.0%	11.5%	5.9%	6.8%	1.9%				
*I feel safe traveling to busier destinations*	Conservative	1.5%	4.6%	9.9%	7.7%	5.0%	14.1	8	0.079	5.7
	Independent	5.6%	9.0%	9.9%	9.0%	2.8%				
	Liberal	5.3%	8.4%	10.8%	7.7%	2.8%				
*I would tend to visit businesses knowing employees were vaccinated more so than other businesses whose employees are not vaccinated*	Conservative^a^	5.6%	4.0%	8.1%	9.3%	1.6%	43.4	8	<0.001	16.7
	Independent^b^	3.4%	4.7%	11.2%	12.7%	4.3%				
	Liberal^ab^	1.9%	2.2%	6.5%	12.7%	11.8%				
*Traveling to areas that have higher rates of COVID-19 vaccinations makes me feel safer*	Conservative	5.9%	3.1%	9.6%	8.4%	1.9%	35.6	8	<0.001	11.4
	Independent^b^	6.8%	3.1%	12.4%	9.6%	4.3%				
	Liberal^b^	3.4%	2.8%	5.9%	11.8%	11.1%				
*Traveling to areas that implement COVID-19 precautions*, *such as mask wearing in restaurants*, *makes me feel safer*	Conservative^a^	4.0%	3.1%	9.9%	6.2%	5.6%	44.1	8	<0.001	21.3
	Independent^b^	2.2%	2.5%	9.0%	13.9%	8.7%				
	Liberal^ab^	0.9%	0.0%	6.2%	12.4%	15.5%				
*Traveling to nature-based destinations (such as a park) makes me feel safe during the pandemic*	Conservative	1.2%	1.9%	9.3%	9.6%	6.8%	16.0	8	<0.001	6.1
	Independent	1.2%	1.5%	9.9%	16.4%	7.1%				
	Liberal	0.3%	1.2%	5.6%	16.4%	11.5%				
*Traveling to rural destinations makes me feel safe during the pandemic*	Conservative	1.9%	1.2%	10.8%	8.7%	6.2%	16.7	8	0.034	5.7
	Independent	1.5%	2.2%	13.6%	13.9%	5.0%				
	Liberal	0.3%	0.9%	10.5%	12.1%	11.1%				

^ab^Statistical difference at a p < 0.001 based on post hoc analyses.

Results also show associations between political affiliation and choosing a destination type and observing ongoing COVID-19 protocols. There were statistically significant differences between conservatives and liberals, and liberals and independents when evaluating areas that implement COVID-19 tactics, such as mask wearing (*F* = 21.3, p < 0.001), having higher vaccination rates (*F* = 11.4, p < 0.001), and having higher vaccination rates within places of business (*F* = 16.7, p < 0.001). Further, variations were also existent when considering the type of location where travel would occur. Specifically, nature-based (e.g., parks; χ^2^ = 16.0, p = 0.043) and rural destinations (χ^2^ = 16.7, p = 0.034) were highlighted as preferred travel locations by all groups, but especially those who identified as independent and liberal, while conservatives conveyed a smaller likelihood to select these locations when choosing a destination type.

## Discussion

The research presented in this paper quantitatively verify two theories about the United States response to COVID-19 in the early stages of the outbreak. First, policy across states was strongly influenced by economic and political motivations, and scientifically driven measures of risk such as climate, population age, and population density were of lower priority [72, 73]. Second, regions of the United States with strong influences to treat COVID-19 as low risk instead suffered higher transmission rates than regions that nominally may have faced greater danger [41, 74].

Our statistical analysis of time-evolving *R*_*t*_ values corroborate commonly held narratives, in the media and the scientific community, of how COVID-19 outbreaks were spatially distributed throughout 2020 [16, 50, 75, 76]. It is not known for certain where COVID-19 was first introduced in the United States, but the first major outbreak was in Seattle in late February, and it proceeded to spread to other urban centers with major airports and throughout the west coast [77, 78]. At this time, it was poorly understood how COVID-19 was spread, masks were not widely available, and ineffective measures such as sanitizing surfaces were deployed [79–81]. Even the most cautious state governments did not begin to declare states of emergency until the latter half of March, several weeks after the virus was introduced. In a sense, the dynamics of the COVID-19 epidemic initially behaved much like an idealized metapopulation Susceptible-Infectious-Recovered model. Incidence curves were exponential everywhere that COVID-19 was present, and reproduction rates among counties were strongly correlated with population density [64, 82].

It was not until late March that masks were widely distributed and serious efforts were made to reduce local *R*_*t*_ values to below one, the threshold for an epidemic outbreak. By this point, the public perception of COVID-19 risk was polarized based on approval of President Trump, and local governments had to strike a balance between effectively controlling the outbreak and protecting their economy from further harm [83, 84]. Most industries were badly hurt by the pandemic, but none more so than Leisure and Hospitality, which accounted for almost 40% of all jobs lost in 2020 [85]. The economic damage of the lost tourism economy was especially felt in rural counties, who are typically reliant on a lower diversity of industries [76].

By early May, the beginning of tourism season in much of the United States, a broad consensus emerged to prioritize economic growth over public health in rural areas with high support for President Trump [86, 87]. Not shown in Fig 5, there was a strong correlation between *R*_*t*_ values and the interaction term between Rurality and Tourism throughout the spring and summer (p < 0.01). The difference between *R*_*t*_ values in rural and urban counties was small but statistically meaningful, and it persisted throughout most of the summer and fall. The relationship between tourism and *R*_*t*_ dissipated by August, but rurality and Trump support were strong predictors until October. In the winter of 2020, individuals throughout the country were more likely to stay at home, and holiday travel resulted in COVID-19 spikes in urban counties [88]. At this point there was no significant difference in *R*_*t*_ between urban and rural counties.

Focusing on the state of Maine, we observed similar dynamics but on a delayed time scale. The t-test scores were not as strong as on the national level, since comparing only 16 counties instead of over 3000 reduces the power of the statistical test. However, the impacts of state policy on *R*_*t*_ values are clearly apparent from the results in Fig 6. In May and June, when tourism and non-essential businesses were closed, there was no significant difference in *R*_*t*_ values between counties [89]. This changed when businesses reopened at the end of June. Almost immediately, we observe *R*_*t*_ values rise in counties with strong Trump support, as they were politically incentivized to patronize businesses and support their local economy [90, 91]. In August, when the number of visiting tourists reached its peak, we observe significantly higher *R*_*t*_ values in rural counties and in counties with large tourism industries [92]. Due to the smaller number of data points compared to the nationwide model, the p-values of these relationships ranged between 1% and 10%, suggesting a moderate correlation but opening the door to other interpretations. This relationship returns to zero at Labor Day, the traditional end of tourism season in Maine, and throughout the rest of 2020 there is no significant difference in *R*_*t*_ between Maine counties [93].

These results are further supported by the presented survey results on social demographics and risk perceptions regarding COVID-19 and travel. Overall, differences across political affiliation groups (i.e., conservative, independent, and liberal) were observed in terms of perceived risk of COVID-19, trust held in sources distributing information regarding the pandemic, and resulting tourism-based decisions (i.e., completion of travel, participating in health and safety protocols in proximity to others, destination selection, etc.). Therefore, the role of political affiliation was found to be a core element in the travel decision-making process of tourists to Maine. Differentiations were predominantly displayed between conservatives and liberals. Conservatives’ perceived risk of COVID-19 and engagement in health preventive behaviors (e.g., mask wearing, social distancing, vaccination planning and reception, etc.) varied from independents and most notably, liberals. Further, information they consulted before travelling (e.g., diverse perspectives of government- and scientific-based sources) and travel-based behaviors pursued, such as seeking out destinations which have high vaccination rates (e.g., the community at large, personnel within local businesses specifically, etc.), differed across these groups. While conservatives displayed contrasting perceptions and behaviors concerning COVID-19 in comparison to independents and liberals, similarities were observed amongst the groups in the decreased level of trust they held in social and news media sources and their selection of nature-based destinations when engaging in tourism procedures.

The results of this manuscript display similar themes found within previous research focused on political affiliation and the pandemic, especially regarding the variation in how each political affiliation acquired and used information or messaging surrounding COVID-19, except for destination selection attributes [31–33]. Considering the heterogeneity in perceptions and behaviors, these differences reflect the pervasive manner intrinsic influences can pose within viewpoints regarding COVID-19 and tourism processes which are followed [24, 25, 29–31, 39]. Due to the evident role of political affiliation, specific implications, such as the pursuit of continued understanding regarding this variable, include facilitating experiences which 1) anticipate an array of tourist perspectives (e.g., varying political-based viewpoints), 2) develop informational resources (e.g., marketing campaigns through print or media sources, information dissemination through app- or portal-based resources, developing cohesive signage to post in accessible and frequented locations, etc.) in alignment with associated perspectives to perpetuate behaviors following public health and safety protocols to increase safe, tourism moments (e.g., distributed messaging focused on potential diverging perspectives to encourage collaboration, management decisions focused on enhancing tourism opportunities for both in- and out-of-state residents to increase revenue and health-based experiences, etc.), and 3) bring forth positive, financial impacts within local economies (i.e., rural tourism destinations in Maine) due to the recognition and engagement of this knowledge to intentionally motivate cognizant travel experiences (e.g., promoting health-based travel opportunities, such as vehicular travel or choosing nature-based destinations, etc.; [30, 31, 33, 39]). Gaining a deeper understanding of the factors that determine tourist travel decision making and behaviors could aid destinations in creating better strategic practices and refine the type and degree of resource allocation needed during uncertain situations, such as the one presented by the pandemic [22, 29, 30].

With the importance of anticipating multifaceted perspectives and resulting behaviors, the modifications which rural tourism destinations implement are not only significant in their requirement, but in the resources needed to ensure the livelihood of all those directly and/or indirectly are addressed [30]. Specifically, in conjunction with implications referenced above, the need for dynamic collaboration and support are constantly integral but undergo an elevation of significance during obscure situations (i.e., COVID-19). Therefore, the formation of engaging, continuous, and proactive partnerships is paramount to ensure rural tourism destinations have optimal resources to solidify their viability and further amplify the success of local tourism-based operations [22, 23, 30]. Through lasting partnerships, support concerning resource availability, the provision of knowledge regarding modern management techniques (e.g., newly adapted or developed procedures in coordination with the pandemic), and genuine moments of connections amongst individuals with shared experiences (e.g., discussion across tourism destinations, affiliated businesses, governmental authorities, etc.) could abound. Therefore, the facilitation and maintenance of such bonds could be resounding in the impact which arises regarding increasing a rural tourism destination’s resiliency overall, especially in the face of the “new normal” which continues to evolve [22, 27, 30, 32].

Concerning the findings, it is important to not only consider the potential influence of political affiliation, but also to investigate the varied factors which could contribute to the formation of an individual’s COVID-19 risk perceptions. Specifically, sociodemographic variables involving age, gender, race/ethnicity, level of education, income, etc. are significant, complex factors which could foster a compound effect and therefore contribute to the viewpoint which an individual maintains toward COVID-19 and associated individual- and societal-based implications [94–96]. For example, Pasion et al. (2020) found that age brought forth impacts in the types and degrees of risk expressed by individuals ranging from young to older-adult groupings regarding COVID-19. Specifically, Pasion et al. (2020) found that anxiety surrounding outcomes of COVID-19 (e.g., short- and long-term health impacts, mortality, etc.) and involved health and safety protocols, such as increased isolation and loneliness, were notable considerations within the risk perceptions of those who were within the middle-aged groupings and younger. Zajacova et al. (2020) similarly focused on the influence of age in consideration of exhibited behaviors during the progression of COVID-19 and how individual actions concerning health evolved. In particular, those within a younger age demographic reported increases in negative health-based behaviors, such as time of exposure to technology, especially during the initial phases of COVID-19 in 2020 when increased social distancing measures were implemented. Changes in behavior were not only detected in consideration of age, but other sociodemographic variables as well. Papageorge et al. (2021) found that income produced inhibiting outcomes in individuals’ ability and probability of completing health and safety measures based on their reduced opportunity to make multiple changes within a condensed time period and transition their day-to-day commitments (e.g., reduced opportunity to work through a remote or hybrid format). Therefore, from age to income and overall alterations the global impact COVID-19 formed, it is significant to reflect other influences (i.e., sociodemographics) in conjunction with political affiliation to further understand the infiltrating, diverse impacts introduced in the lives of many [94–97]. This is imperative to not only ensure the livelihood and longevity of all is revered as being of the utmost importance, but to continuously work through intentional measures (e.g., policy development, increasing partnerships and support on multiple scales, etc.) to reduce the introduction and proliferation of negative, inequitable, and unequal influences as well.

The results of this paper imply that local attitudes towards COVID-19 were influenced as much by economic and political motivations as by scientifically driven risk estimates and strategies. This is true both on the governmental level, in terms of enacting stay-at-home orders, business closures, and travel restrictions, and on the individual level, in terms of the personal choice to wear a mask or avoid public spaces [98–100]. Paradoxically, erroneously low perceptions of COVID-19 risk are linked to higher transmission rates and a greater number of incidence cases [16, 101]. Correlation does not imply causation, therefore one should be careful not to infer too much about the effects of specific policy choices on mitigating or encouraging the spread of COVID-19 in specific states and counties. However, it would be prudent to prepare and respond to future pandemics by focusing on metrics of disease risk determined by the consensus of the scientific community [13, 63].

## Conclusion

This manuscript statistically verifies common narratives regarding public attitudes towards the introduction of COVID-19, risk perceptions of the epidemic among various socioeconomic groups, and how those attitudes are directly correlated with the severity of the epidemic. This research also demonstrates the value of studying spatially and temporally varying *R*_*t*_ values during the early phase of an epidemic outbreak, rather than focusing only on the number of incidence cases per capita. Our research focuses only on the early outbreak phase of the COVID-19 epidemic in the United States so that predictor variables were focused on choices in human behavior. A further analysis that proceeds into 2021 and 2022 could elucidate the effects of vaccine hesitancy and the arrival of new variants on *R*_*t*_ variability. This research could also help inform local policy makers on determining an optimal balance between economic considerations and public health for future epidemics.
