# Correction: Risk perceptions and behaviors concerning rural tourism and economic-political drivers of COVID-19 policy in 2020

**DOI:** 10.1371/journal.pone.0334027

**Published:** 2025-10-07

**Authors:** The PLOS One Staff

The images for Figs 1 and 2 are incorrectly switched. The image that appears as Fig 1 should be Fig 2, and the image that appears as Fig 2 should be Fig 1. The figure captions appear in the correct order. The authors have provided a corrected version of figures here.

The publisher apologizes for the errors.

**Fig 1:**
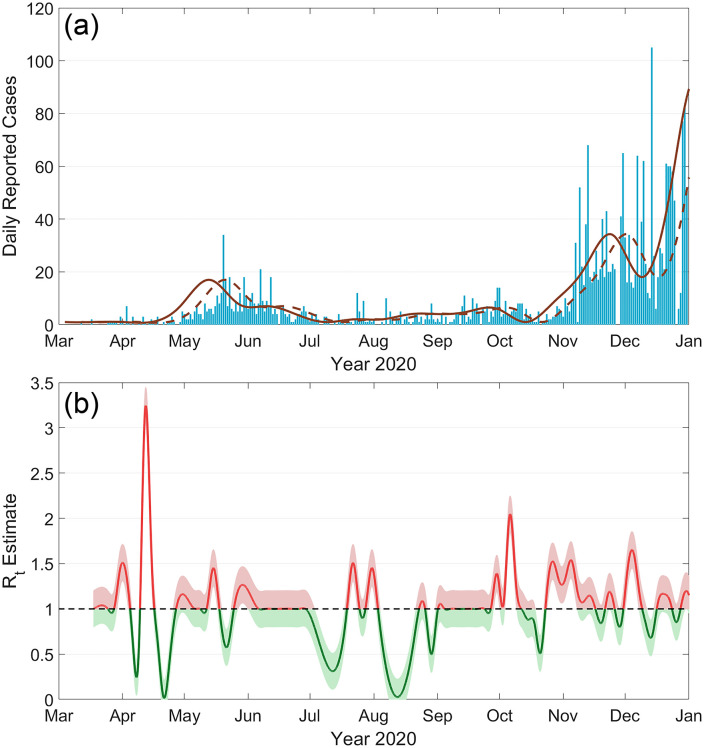
(a) A bar graph of the number of reported cases of COVID-19 in Kennebec County, Maine, throughout 2020. The raw case data was compiled from a variety of sources by the New York Times. The data is cleaned, first by averaging the data over a seven-day period (dotted line), then by assuming an 8-day lag between the onset of infection and the appearance of symptoms (solid line). (b) The effective reproduction number (Rt) in Kennebec county, computed from the cleaned case data using the Bettencourt and Ribeiro method. The shaded region represents a 90% confidence interval. https://doi.org/10.1371/journal.pone.0299841.g001.

**Fig 2:**
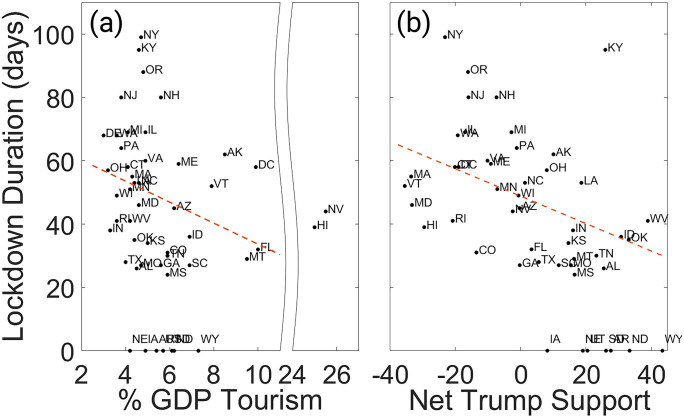
Scatter plots showing the duration of lockdown orders of all 50 states and District of Colombia, compared to their % GDP tourism in 2019 and margin of incumbent President Trump’s election victory in November 2020. Two states (Hawaii and Nevada) are considered outliers and are not included in the linear model fit, and two states (California and New Mexico) had lockdown durations that lie far above the upper boundary of the plots. Seven states which did not enact lockdown orders (Arkansas, Iowa, Nebraska, North Dakota, South Dakota, Utah, and Wyoming) are recorded with a duration of 0 days. https://doi.org/10.1371/journal.pone.0299841.g002.
